# Physical Activity in Patients with Prader-Willi Syndrome—A Systematic Review of Observational and Interventional Studies

**DOI:** 10.3390/jcm10112528

**Published:** 2021-06-07

**Authors:** Alice Bellicha, Muriel Coupaye, Héléna Mosbah, Maithé Tauber, Jean-Michel Oppert, Christine Poitou

**Affiliations:** 1 INSERM, Nutrition and Obesities: Systemic Approaches (NutriOmics), Research Unit, Sorbonne University, F-75013 Paris, France; alice.bellicha@u-pec.fr; 2 Assistance Publique-Hôpitaux de Paris (AP-HP), Pitié-Salpêrière Hospital, Department of Nutrition, Rare Diseases Center of Reference ‘Prader-Willi Syndrome and Obesity with Eating Disorders’ (PRADORT), Sorbonne University, F-75013 Paris, France; muriel.coupaye@aphp.fr (M.C.); helena.mosbah@aphp.fr (H.M.); jean-michel.oppert@aphp.fr (J.-M.O.); 3 Centre de Référence du Syndrome de Prader-Willi, Hôpital des Enfants, Axe Pédiatrique du CIC 9302/INSERM, Hôpital des Enfants, Institut Toulousain des Maladies Infectieuses et Inflammatoires, INSERM UMR1291, CNRS UMR5051, Université Toulouse III, F-31000 Toulouse, France; tauber.mt@chu-toulouse.fr

**Keywords:** Prader-Willi syndrome, syndromic obesities, physical activity, sedentary time, implementation, systematic review

## Abstract

Physical activity (PA) is an important aspect of the management of patients with Prader-Willi syndrome (PWS). However, the day-to-day implementation of PA programs is particularly challenging in these patients. This systematic review aimed (1) to describe habitual PA and sedentary behavior and (2) to assess the effects of PA interventions and to describe their implementation process, in children and adults with PWS. A systematic search of controlled trials, single-group interventions, observational, and qualitative studies published up to December 2020 was performed. Twenty-five studies were included. Habitual PA was found to be lower in patients with PWS compared to controls without obesity or with non-syndromic obesity. Habitual PA was positively associated with lean body mass and bone parameters in children with PWS, and these finding were strengthened by intervention studies reporting an increase in both outcomes after a PA program. PA programs also improved physical function (muscle strength, walking distance, and coordination), without significant effect on weight and fat mass. Attendance to exercise sessions was usually high and no serious adverse effect was reported. In conclusion, supervised PA programs are beneficial for children and adults with PWS. Support should be provided to families to facilitate their implementation in real-life settings.

## 1. Introduction

Prader-Willi syndrome (PWS) is the most frequent cause of genetic obesity [1], with a prevalence between one in 20,000 and one in 30,000 births [2]. PWS is a complex genetic neurodevelopmental disorder caused by an absence of expression of imprinted alleles of paternal origin on chromosome 15 [1]. PWS is characterized by severe hypotonia and feeding difficulties in early infancy, followed in early childhood by excessive eating and gradual development of severe obesity [1]. Patients with PWS display low to moderate intellectual disability, as well as decreased motor competencies and physical fitness [1,3]. In adulthood, patients with PWS are prone to develop severe complications, such as cardiac or respiratory failure as well as various comorbidities such as scoliosis [4].

Promoting physical activity (PA) is an important objective of the management of PWS in both children and adults [5]. However, less than 10% of children with PWS reach the recommended level of PA [6,7], and this proportion does not exceed 20% in adults [8,9]. Over the last 10 years, a number of studies have assessed habitual PA and, to a lesser extent, sedentary behavior in these patients [7,8,9,10,11]. Most studies used objective methods such as accelerometers that are considered as the method of reference for measuring PA and sedentary time, especially in patients with cognitive impairment for whom self-reporting may be particularly challenging [12]. If most studies have reported a low level of PA in patients with PWS, it is not clear whether the decreased PA is related to obesity per se, or to the physical and intellectual disabilities associated with PWS. It is therefore relevant to synthetize the available literature comparing habitual PA and sedentary behavior in patients with PWS and in controls without obesity, with non-syndromic obesity or with other neurodevelopmental disorders.

A recent systematic review reported beneficial effects of PA interventions, mostly in the forms of supervised exercise training programs, in patients with PWS [3]. The benefits were mainly related to improved physical fitness, while the effects on body composition were not consistent [3]. Given the relatively few studies included and their diversity in terms of population, interventions, and outcomes assessed, this review did not provide a quantitative analysis of findings. Since then, several studies were published [8,13,14,15,16], adding to the body of evidence on the effect of PA interventions in patients with PWS. Importantly, some of these studies described in detail the implementation process of the PA intervention [8,13,16], which is of great importance to facilitate their transferability in real-life settings.

Therefore, the first aim of this systematic review was to describe habitual PA and sedentary behavior in children and adults with PWS and to analyze their relations with body composition and health status. The second aim was to update the evidence on the effects of PA interventions in these patients and to describe their implementation process.

## 2. Methods

This systematic review follows the Preferred Reporting Items for Systematic Reviews (PRISMA) guidelines and is registered in the PROSPERO database (registration number: CRD42021224978).

### 2.1. Search Strategy

Three electronic databases (PubMed, Web of Science, and EMBASE) were searched for original articles published between January 2000 and December 2020 using the search terms “Prader-Willi Syndrome” AND “Physical activit*” OR “Exercise” OR “Training” OR “Aerobic training” OR “Resistance training” OR “Strength training” OR “Walking” OR “Sitting time” OR “Sedentary”. Reference lists from the resulting reviews and articles were screened to identify additional articles.

### 2.2. Study Selection, Inclusion, and Exclusion Criteria

Articles were included if they involved children and adolescents (2–18 years) or adults (≥18 years) with genetically confirmed PWS. Although most patients with PWS present with obesity, some children with PWS have normal weight [17]. Therefore, body mass index (BMI) was not defined as an inclusion criterion.

For study aim #1, studies were included if (1) PA or sedentary behavior was assessed using either self-report or device-based (pedometers, accelerometers) methods (2) in a group of children and/or adults with PWS. For study aim #2, studies were included if (1) children and/or adults with PWS participated in a PA intervention (PA alone or combined with a dietary intervention), and (2) the study design was a controlled trial (randomized or not) or a single-group intervention study. Studies describing the implementation process of the intervention were also included. Only studies written in English were included.

### 2.3. Data Extraction and Synthesis

Data were extracted by one author (AB) using standardized forms and then checked by a second author (CP). The characteristics of each article included reference, country, study design and quality, number of participants included in the group of patients with PWS, characteristics of these patients (% female, age, BMI, body fat, genetic diagnosis, information on growth hormone replacement therapy, GHRT), description of PA intervention when relevant, outcomes and assessment methods. Data related to habitual PA and sedentary behavior were reported, along with the effects of PA interventions on body weight and body composition (fat mass, lean body mass), physical function, habitual PA, health-related quality of life and health outcomes.

We adopted a semi-quantitative approach to summarize the findings. To address aim #1, we calculated the number of studies reporting a lower level of habitual PA or sedentary behavior, a higher level or no difference in PA and sedentary behavior in patients with PWS compared to a control group. To address aim #2, we calculated the number of studies reporting a positive, negative or no effect on each pre-specified outcome. Finally, we used a narrative approach to describe the implementation process of interventions.

### 2.4. Quality Assessment

To assess study quality, we used the tool developed by the National Heart, Lung and Blood Institute (NHLBI, Bethesda, MD, USA) that has been previously used for defining guidelines for the management of obesity [18]. The original assessment forms for controlled trials and single-group intervention studies were used. The global rating of study quality was determined based on the number of “fatal flaws” that were identified: good quality (0 fatal flaws), fair quality (1 fatal flaw), or poor quality (≥2 fatal flaws). Three or four assessment items represented fatal flaws if answered “No/Not reported/Can’t determine”: similar baseline characteristics, high adherence, and similar background treatments for controlled trials; eligibility criteria predefined, representativeness of participants, drop-out rate <20% or intent to treat analysis, statistical analyses examined changes in outcomes for single-group interventions. Quality assessment was conducted independently by two reviewers (AB and CP). Any disagreement between the reviewers was resolved through discussion (with a third (HM) author where necessary).

## 3. Results

**Figure 1:**
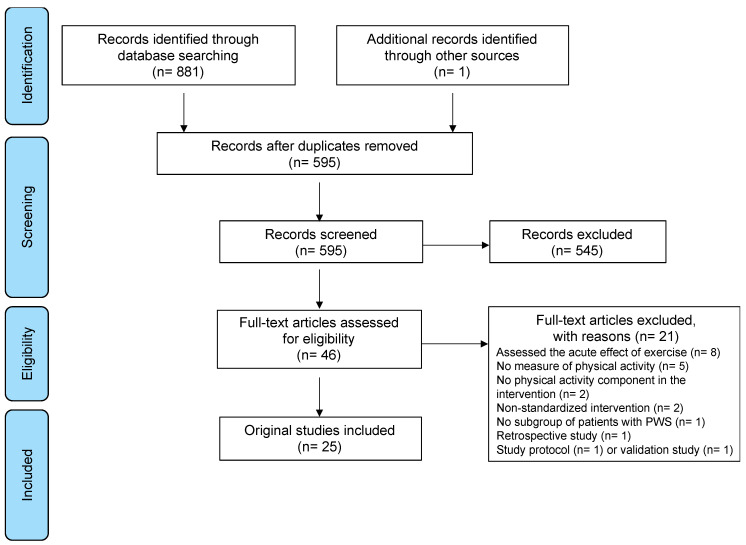
Systematic review flow diagram.

The database search yielded 4348 articles (2858 after removing duplicates), 2793 of which were eliminated based on titles and abstracts alone (Figure 1). The full text was retrieved from 46 articles and 25 met the inclusion criteria.

### 3.1. Study Characteristics and Quality

Seven studies were published between 2000 and 2009 and the remaining 18 studies were published between 2010 and 2020. Studies were conducted in the USA (N = 11 studies), the Netherlands, Italy, and Australia (N = 3 each), Switzerland (N = 2), France, Norway, and Taiwan (N = 1 each) (Table 1). Children and/or adolescents were included in 13 studies, adults in seven studies and both children/adolescents and adults in five studies. Both males and females were included in all but one study with only females [8]. The median (min–max) sample size of participants with PWS was 20 (6–123). Information on GHRT was reported in 14 studies. Three studies included only patients who had never received or were not currently receiving GHRT. In the remaining studies, most patients had previously received or were currently receiving GHRT. Eleven studies were observational, and 14 studies were interventional. In these intervention studies, eight distinct PA interventions were assessed in one randomized controlled trial (RCT) [14], four non-randomized controlled trials [19,20,21,22] and three single-group intervention studies [8,23,24]. Six additional studies reported the effect of these eight interventions [13,15,16,25,26,27]. Among the five randomized or non-randomized controlled trials, two were rated as good-quality trials [14,21], two as a fair-quality trial [19,20], and two as poor-quality trials [22]. Among the three single-group intervention studies, two were rated as fair-quality studies [8,24] and one as a poor-quality study [23].

### 3.2. Description of Habitual PA and Sedentary Behavior in Patients with PWS

Fourteen studies measured habitual PA in patients with PWS with objective or self-reported methods (12 and two studies, respectively). Data from these 14 studies are presented in detail in Table S2. Data from the nine studies that measured PA with objective methods in a group of patients with PWS and in a control group are summarized in Table 2. Compared to controls with normal weight and to controls with non-syndromic obesity, the volume of PA was lower in patients with PWS in two (100%) and four (100%) studies, respectively. Compared to controls with non-syndromic obesity, the duration of light-intensity PA was lower in children and/or adults with PWS in two (100%) studies, and the duration of moderate-to-vigorous PA was lower in one (25%) study and tended to be lower in three (75%) studies. Total sedentary time and sedentary time spent in prolonged uninterrupted bouts (≥30 min) was higher in adults with PWS in one (50%) study. The proportion of patients with PWS meeting PA guidelines was between 5 and 20% of children and adults, respectively, and did not significantly differ from controls with non-syndromic obesity. Finally, PA-induced energy expenditure was lower in children with PWS compared to children with non-syndromic obesity, whether expressed as absolute value or relative to body weight [29].

### 3.3. Relations between Habitual PA and Health Outcomes

Objectively measured habitual PA was found to be positively associated with lean body mass in children and adults with PWS [22,29] and with bone parameters (hip and body bone mineral content and bone mineral density) in children with PWS [11]. No significant association was reported with body fat in either children or adults with PWS [7,10,31,32]. Reported participation in sports and PA was not found to be associated with physical function in children with PWS [28] but a trend towards an association between daily steps and walking capacity (*p* = 0.13) was reported in adults with PWS [10]. Finally, in children with PWS, no significant association was found between habitual PA and a composite score of metabolic syndrome [7], markers of inflammation [7], behavior and emotional problems [28], or the degree of intellectual disability [28].

### 3.4. Effectiveness of PA Interventions in Patients with PWS

#### 3.4.1. Description of Interventions

A total of eight distinct interventions were assessed among the 14 intervention studies included in this review. A PA program was conducted alone in six studies [8,14,20,21,22,24] and in combination with a dietary intervention (i.e., hypocaloric diet) in two studies [19,23]. The PA program was conducted at home in four studies [8,20,21,22], in a hospital or institutional setting in two studies [19,23], and in a community gymnasium in one study [14] (setting not reported in one study [24]). Resistance training was performed in five studies [14,19,20,22,24], aerobic training in one study [23], aerobic training in the form of playground and active video games in one study [21], and combined aerobic and resistance training in one study [8]. The duration of the program ranged from 10 weeks [14,22] to six months [19,21]. In one study, the program was conducted during four weeks and repeated up to four times per year [23]. The weekly number of sessions ranged from one [22,24] to seven [20] and daily duration of exercise ranged from 4 min [22,24] to ≥6 h [23]. PA was supervised by PA instructors or physiotherapists in three studies [8,13,24], by parents in three studies [20,22,26] or was unsupervised in one study [19] (supervision not reported in one study [23]).

#### 3.4.2. Effect of Interventions on Health Outcomes Status

Six (86%) studies reported no significant effect of PA interventions on body weight and body fat in children and adults with PWS, as shown in Table 3. The only study reporting a significant weight loss included both a dietary intervention and PA program based on more than 6 h/d of aerobic exercise for four weeks, up to four times per year. Three (100%) studies conducted in children reported an increase in lean body mass and one (100%) study reported an increase in bone mineral density after a 2.5- to 6-month PA program. In adults, two (100%) studies reported no significant effect of exercise on lean body mass. Five (71%) studies found a significant improvement in physical function (muscle strength, walking distance, and coordination) after the PA program in children and adults with PWS. Habitual PA increased after the PA program in three (60%) studies, but sedentary time was unchanged in three (100%) studies. Finally, two (100%) reported no change in cardiometabolic markers or in health-related quality of life. Three studies involving only children or adolescents reported that a large majority of participating were on growth hormone (GH) replacement therapy at the time of the study or had been on such therapy for extended periods of time before the study [20,22,26]. Two studies involving both children/adolescents and adults reported that a minority (10 to 25%) of participants were on GH replacement therapy at the time of the study [14,23].

#### 3.4.3. Implementation of Interventions

- Attendance, satisfaction, enjoyment, adverse events

Attendance to prescribed exercise sessions was close to 90% in four studies, whatever the setting of intervention, i.e., home- or institution-based [8,14,19,21]. The only study reporting lower attendance (<80% for most patients) stands apart as patients were encouraged to exercise more than 6h per day [23]. Satisfaction and enjoyment were each assessed in one study [8,16]. Adults with PWS and their family reported a very high level of satisfaction (4.4 and 4.8 on a 5-point scale, respectively) [8] and children with PWS reported moderate-to-high levels of enjoyment (3.1 to 3.9 on a 5-point scale) [16]. No adverse event was reported [8,13] but non-serious events occurred in a few patients, such as a “meltdown” during an exercise session, anxiety leading to skin picking or food taken without permission [13].

- Adaptation of intervention

In the study by Grolla et al., low initial attendance increased after introducing suitable entertainment, sufficient rest periods, psychomotor activities and musicotherapy [23]. In the study by Bellicha et al., a variety of aerobic activities have been set up (e.g., walking, Nordic walking, ball games, racket games, etc.) to make the sessions more enjoyable and to increase motivation [8]. At each session, the choice of aerobic activity was made with involvement of the patient [8]. In the study by Rubin et al., the content of the intervention was tailored to children and included, in addition to strength exercises, playground games using play/sport equipment and active video games [16].

- Supervision

In the study by Shields et al., exercise sessions were conducted by 14 community-based physiotherapists, of whom 11 had no previous experience with patients with PWS [13]. Clear communication, clarity and adaptability were described by physiotherapists as essential skills, as well as the ability to create a positive relation with the patients and to develop their confidence. In our study by Bellicha et al., exercise sessions were conducted by 5 PA instructors who all received specific training on PWS before starting the program [8]. The main difficulties reported by instructors were related to psychological disorders associated with PWS (emotional lability and immaturity, mental rigidity), limited physical function and a lack of motivation. Adaptability to the patient’s motivation, mood and fatigue was also reported as an essential skill [8]. In the study by Rubin et al., exercise sessions were supervised by parents who received significant support throughout the intervention: hands-on training sessions, frequent support calls, printed PA curriculum, or problem-solving sessions, as well as all needed equipment provided for free [16]. Scheduling conflicts and lack of motivation of the child were identified by parents as the main barriers to conduct the PA sessions as planned [16].

## 4. Discussion

This systematic review described the level of habitual PA and sedentary behavior in children and adults with PWS. Findings from 14 studies were synthetized, of which 12 studies provided an objective measure of PA or PA-related energy expenditure using accelerometers [6,7,8,9,10,11,26,31], pedometers [20,22], or indirect calorimetry measures in a metabolic chamber [29,32], and two studies provided a self-reported measure of PA [28,30]. The total volume of PA was consistently found to be lower in patients with PWS compared to patients with non-syndromic obesity [8,26,29,32]. Although we have no explanation for this observation, the deficit in lean body mass in patients with PWS compared to people of similar corpulence may explain, at least in part, the lower level of PA observed in patients with PWS. In addition, although data came from only two studies, patients with PWS appear to spend less time in light-intensity PA and more time in a sedentary position compared to patients with non-syndromic obesity [6,8]. The proportion of patients with PWS who met the public health PA guidelines was very low, estimated between 5% and 8% in children [6,7] and between 15 and 25% in adults [8,9] but, in contrast with previous findings, it was not different from that reported in controls with non-syndromic obesity [7,8,9]. This discrepancy can be explained by the fact that PA volume (e.g., daily steps) considers any intensity of PA (light, moderate, or vigorous), whereas PA guidelines are based on the amount of moderate-to-vigorous PA (MVPA). In the 2020 WHO guidelines on PA and sedentary behavior, children are advised to engage in 60 min/day of MVPA, and adults in 150 to 300 min/week (i.e., at least 30 min/d) of MVPA [34]. They are also advised to limit the amount of time spent being sedentary by engaging in PA of any intensity [34]. Replacing sedentary time by light-intensity PA is indeed increasingly recognized as beneficial for maintaining health status across the lifespan [35]. Interestingly, recent data suggest that the benefit may be higher for individuals with low levels of PA [36], which is most often the case for patients with PWS. Overall, the data included in this review suggest that reaching the recommended level of at least moderate-intensity PA may be particularly challenging for patients with low physical fitness such as patients with PWS [3]. The guidelines based on MVPA should therefore be seen as a goal to strive for rather than a minimum level to be achieved. Promoting light-intensity PA throughout the day may be a complementary and more feasible approach that may bring substantial health benefits in patients with PWS and could represent a first step in progressing towards higher levels of PA.

The second aim of this review was to assess the effects of PA interventions in patients with PWS. Findings from 14 intervention studies, investigating the effect of eight distinct interventions, were synthetized. The most consistent benefit of PA interventions was related to the improvement of physical fitness in both children and adults with PWS. Studies have reported improved walking capacity [8], muscle strength [20,21,24], or gait parameters [19] after a PA program. Such effects are likely to represent important benefits in patients with PWS who typically present with impaired muscle strength, cardiorespiratory fitness, and gait patterns [3]. In contrast, most studies reported no significant effect of PA interventions on weight and fat loss [8,14,22,26,27]. The only study reporting a significant weight loss also included a dietary intervention in addition to several hours of exercise per day, thus preventing the authors from attributing this effect to PA alone [23]. In line with these findings, observational studies reported no significant relation between habitual PA and body fat in either children or adults with PWS [7,10,31,32], which strengthens the conclusion that PA may not have substantial effect on weight loss in patients with PWS. This is in agreement with the known effect of PA in adults with non-syndromic obesity, in whom only a weight loss of small magnitude (2 to 3 kg on average) is observed after an exercise training program [37]. In children with PWS, PA interventions seem to have a beneficial effect on lean body mass [20,22,26] and bone mineral density, which is an important major health benefit in these patients with low baseline values of lean body mass and bone mineral density [15]. Observational studies have also reported a positive relation between habitual PA and lean body mass [22,29] or bone parameters [11]. Importantly, a large majority of children and adults participating in the PA intervention were on GH replacement therapy at the time of the study or had been on such therapy for extended periods of time before the study [15,20,22,26]. In this context, PA is likely to reinforce the effects of GH therapy, and it has therefore been described as a co-adjuvant intervention to GH therapy in children with PWS [3].

Although half of the included studies did not report participation rate, available data show high participation of patients with PWS to prescribed PA sessions, whether the program was conducted at home [8,21], in an institution [19], or in a community setting [14]. All sessions were supervised, which is likely to be a key element in promoting patients’ participation and engagement. Creating a daily routine was found to facilitate sustained participation in the PA program, according to parents and physiotherapists who supervised the sessions [13,16]. Inside this routine, the ability to adapt the PA session according to the patient’s motivation, mood or fatigue was also perceived as an essential skill for supervisors [8,13]. In this sense, the PA program supervised by parents in the study by Rubin et al. [16] was designed to provide occasional opportunity for the child to choose an activity of his/her choice. Interestingly, children gave the highest enjoyment ratings to these activities named “player’s choice” [16]. In this study and in others, emphasis was placed on offering a variety of activities that would make PA as pleasant and motivating as possible: playground games, dance games, active video games in a home-based program for children [16], Nordic walking, ball games, racket games in a home-based program for adults [8], or psychomotor therapy and musicotherapy in a hospital-based program for adults [23]. Note that PA instructors received specific training on PWS before the program in one study [8], and that parents received considerable support in another study [16], in the form hands-on training sessions, frequent support calls, printed PA curriculum, or problem-solving sessions. Overall, these data suggest that a structured routine allowing for daily adaptation should be established to promote PA in children and adults with PWS, which mirrors current recommendations for dietary management in these patients [5]. Families often feel overwhelmed and isolated, and they may need external support and supervision to engage their child in a PA program. The success of PA promotion is likely to depend on the training of PA instructors involved, on the affordability of the PA program, and on the support and guidance provided to families by healthcare professionals.

This systematic review has methodological strengths but also some limitations that should be mentioned, such as the relatively limited number of studies included, the small sample size of most of these studies, and the low methodological quality of about two thirds of intervention studies included. Note, however, that implementing PA interventions is challenging in patients with PWS, especially because PWS is a rare disease with a limited recruitment potential. Patients in whom it is possible to assess PA level with objective methods may also not be representative of the entire population of patients with PWS, especially regarding cognitive and behavioral disorders. Important issues could not be addressed in this review. First, given the low number of interventions assessed and their heterogeneity, we were not able to compare effectiveness across studies. For clinical purposes, it would indeed be important to identify the type of exercise that would be both most feasible and effective in children and adults with PWS. Based on current recommendations for patients with obesity [18], we can expect that a combination of aerobic and strength training would be the most effective in patients with PWS. Second, none of the studies included compared the effectiveness of PA interventions across genders, preventing us from concluding on the important question of gender differences. Moreover, published studies did not assess whether PA can help prevent weight gain over time. In particular, preventing weight gain at some critical periods, such as transition from childhood to adulthood, would be an important benefit. Finally, very little is known on the effects of PA on metabolic health in patients with PWS, although PA is recognized as highly beneficial for such outcomes in patients with common forms of obesity [38]. Considering that type 2 diabetes is a frequent comorbidity in adults with PWS [39,40], there is a need to better understand how PA could prevent the risk of type 2 diabetes in these patients and can contribute to its management.

## 5. Conclusions

Patients with PWS spend less time in light-intensity PA, more time in sedentary occupations, and tend to spend less time in moderate-to-vigorous PA compared to patients with non-syndromic obesity, which results in a lower total volume of PA. Supervised PA programs are feasible in both children and adults with PWS and may provide several benefits related to improved physical function and, in children only, increased lean body mass and bone mineral density. Importantly, these benefits occur even in the absence of weight and fat loss. To facilitate the implementation of PA programs in real-life settings, PA sessions should be supervised by trained PA instructors or by parents provided they receive significant support and guidance on how to conduct and adapt the program. PA programs should be developed jointly by care teams specializing in the management of PWS, by PA instructors, and by parents and professionals involved in the day-to-day education and care of these patients. As for all patients with obesity, but even more so for patients with PWS, the PA program should be individually tailored and should offer a variety of activities that are as enjoyable as possible.

## Figures and Tables

**Table 1:** Study characteristics (N = 25 studies).

**Observational Studies**				
**Reference (Country)**	**Study Design and Quality**	**Characteristics of Patients with PWS**	**Characteristics of the Control Group**	**Outcomes of Interest (Methods)**
Borland 2020 [28] (Australia)	Comparative cross-sectional study	Adults with PWS (N = 30, 11 F + 19 M) Data are mean (SD) (min–max) Age, year: 30.9 (7.8) (18–46) GHRT: not reported	Adults with Down Syndrome (N = 64, 39 F + 25 M) Age, year: 27.9 (4.5) (20–36) General population (N = 316, 133 F + 183) Age, year: 28.8 (4.5) (19–39)	- Sports and PA participation (question from the Australian Bureau of Statistics General Society survey) - Self-reported physical mobility (item from the Index of Social Competence)
Butler 2007 [29] (USA)	Comparative cross-sectional study	Children/adults with PWS (N = 48, 27 F + 21 M) Data are mean (SD) (min–max) Age, year: 23 (9) (10–45) BMI, kg/m^2^: 34 (9) Body fat, %: 51 (8) Deletion, n (%): 27 (56%) Uniparental disomy: 21 (44%) Type 2 diabetes, n (%): 8 (17%) Not currently on GHRT (N = 48)	Children/adults with NSO (N = 24, 15 F + 9 M) Age, year: 27 (13) (11–49) BMI, kg/m^2^: 41 (8) Body fat, %: 50 (7) Type 2 diabetes, n (%): 3 (13%)	- Body weight and composition (DXA) - Total EE, resting EE, standing EE, PAEE (calorimetric chamber)
Castner 2014 [6] (USA)	Comparative cross-sectional study	Children with PWS (N = 24, 12 F + 12 M) Data are mean (SD) (min–max) Age, year: 11.2 (2.3) (8–16) BMI, kg/m^2^: 29.4 (12.7) Body fat, %: 45.8 (11.0) Deletion, n (%): 10 (42%) Uniparental disomy: 3 (13%) Imprinting defect: 3 (13%) Unknown subtype, n (%): 8 (33%) Type 2 diabetes: 1 (4.2%) Currently on GHRT (N = 15), had previously been on GHRT (N = 6)	Children with NSO (N = 40, 19 F + 21 M) Age, year: 9.8 (1.1) (8–11) BMI, kg/m^2^: 27.3 (4.0) Body fat, %: 44.1 (5.7)	- Body weight and composition (DXA) - Habitual PA (accelerometer)
Duran 2016 [11] (USA)	Cross-sectional study	Children with PWS (N = 23, 12 F + 11 M) Data are mean (SD) (min–max) Age, year: 11.0 (2.0) (8–14) BMI, kg/m^2^: 29.4 (13.0) Body fat, %: 45.9 (11.2) Deletion, n (%): 10 (43%) Uniparental disomy, n (%): 3 (13%) Unknown subtype, n (%): 10 (43%) Currently on GHRT (N = 15), had previously been on GHRT (N = 5), had never been on GHRT (N = 3)	--	- Body weight, body composition, bone mineral density (DXA) - Habitual PA (accelerometer)
McAlister 2018 [7] (USA)	Comparative cross-sectional study	Children with PWS (N = 21, 12 F + 9 M) Data are mean (SD) (min–max) Age, year: 10.7 (2.6) (8–15) BMI, kg/m^2^: 28.2 (10.0) Body fat, %: 46.0 (8.9) Deletion, n (%): 9 (43%) Uniparental disomy, n (%): 5 (24%) Unknown subtype, n (%): 7 (33%) Currently on GHRT (N = 16)	Children with NSO (N = 34, 17 F + 17 M) Age, year: 9.6 (1.0) (8–15) BMI, kg/m^2^: 29.0 (5.1) Body fat, %: 45.4 (6.4)	- Habitual PA (pedometer) - Glucose metabolism (fasting glucose, HOMA-IR), lipids (TG, HDL), blood pressure
Nordstrom 2013 [9] (Norway)	Comparative cross-sectional study	Adults with PWS (N = 22, 13 F + 9 M) Data are mean (SD) Age, year: 28.1 (7.5) BMI, kg/m^2^: 30.7 (6.2) Deletion, n (%): 15 (68%) Uniparental disomy, n (%): 5 (23%) Unknown subtype, n (%): 1 (5%) Non-genetically confirmed: 1 (5%) GHRT: not reported	Adults with Down Syndrome (N = 40, 25 F + 15 M) Age, year: 26.8 (7.5) BMI, kg/m^2^: 31.8 (6.5) Adults with Williams syndrome (N = 25, 16 F + 9 M) Age, y: 31.5 (6.2) BMI, kg/m^2^: 26.6 (6.5)	- Body weight - Habitual PA (accelerometer) - Physical function (6MWT)
Sellinger 2006 [30] (USA)	Comparative cross-sectional study	Children/adults with PWS (N = 29, 11 F + 18 M) Data are mean (SD) Age, year: 16.8 (7.0) GHRT: not reported	Children/adults with Down Syndrome (N = 104, 38 F + 66 M) Age, year: 17.0 (9.9) Children/adults with Williams Syndrome (N= 90, 48 F + 42 M) Age, y: 14.2 (9.6)	- Leisure activities, including physical activities (Leisure Activities Questionnaire)
van den Berg-Emons 2008 [31] (the Netherlands)	Comparative cross-sectional study	Children with PWS (N = 12, 7 F + 5 M) Data are mean (SD) Age, year: 11.4 (2.4) (7–16) Body fat, %: 46.4 (6.7) (30.1–52.5) All children were enrolled in a trial assessing the effect of GHRT	Children without obesity (N = 12, 7 F + 5 M) Age, year: 11.1 (2.1) [8,9,10,11,12,13,14,15,16]	- Body weight - Habitual PA (accelerometer)
Van Mil 2000 [32](the Netherlands)	Comparative cross-sectional study	Children with PWS (N = 17, 10 F + 7 M) Data are mean (SD) (min–max) Age, year: 11.9 (3.4) (7–19) BMI, kg/m^2^: 23.5 (6.0) (15.2–38.1) Body fat, %: 43.7 (7.9) (29.4–59.5) Had never been on GHRT (N = 17)	Children with NSO (N = 17, 10 F + 7 M) Age, year: 11.3 (2.6) (6–15) BMI, kg/m^2^: 26.0 (6.5) (13.5–39.4) Body fat, %: 39.1 (8.8) (16.3–46.7)	- Body composition (deuterium dilution) - Basal metabolic rate, PA energy expenditure (metabolic chamber)
Van Mil 2001 [33] (the Netherlands)	Same design as [32]	Same participants as [32] Had never been on GHRT (N = 17)	Same intervention as [32]	- Body composition (deuterium dilution), bone mineral density (DXA) - Basal metabolic rate, PA energy expenditure (metabolic chamber)
Woods 2018 [10] (USA)	Cross-sectional study	Adults with PWS (N = 19, 8 F + 11 M) Data are mean (SEM) (min–max) Age, year: 34.5 (4.3) (18–62) BMI, kg/m^2^: 26.7 (1.3) (19.5–35.0) Body fat, %: 26.8 (1.7) (16.6–41.9) GHRT: not reported	--	- Body weight and composition (bioelectrical impedance) - Habitual PA (accelerometer) - Physical function (6MWT)
**Intervention Studies**				
**Reference (Country)**	**Study Design and Quality**	**Characteristics of Patients with PWS Participating to the PA Intervention**	**Description of Intervention**	**Outcomes (Method)**
Bellicha 2020 [8] (France)	Single-group intervention (control group for baseline measures) Study quality: fair	Adults with PWS (N = 10 F) Data are median (P25–P75) (min–max) Age, year: 28.8 (24.2; 33.0) (19–48) BMI, kg/m^2^: 37.2 (34.3; 45.8) (31.8–52.8) Body fat, %: 51.9 (49.2; 54.7) (41.1–62.4) Deletion: 9 (90%), Uniparental disomy: 1 (10%) Type 2 diabetes: 2 (20%) Had previously been on GHRT (N = 4), had never been on GHRT (N = 6)	- PA program with no concurrent dietary intervention - Setting: home-based program - Program duration: 16 weeks - Frequency: 2 sessions/week of 60 min - Type: aerobic + resistance training - Supervision: total (PA instructor)	- Body weight and composition (DXA) - Habitual PA and sedentary time (accelerometer) - Physical function (6MWT, handgrip strength, stance test) - Health-related quality of life (SF-12)
Eiholzer 2003 [20] (Switzerland)	NRCT Study quality: poor	Children with PWS (N = 17, 8 F + 9 M) Data are mean (min–max) Age, year: 10.5 (4–18) Currently or previously on GHRT for at least 3 years (N = 17)	- PA program with no concurrent dietary intervention - Setting: home-based program - Program duration: 12 weeks - Frequency: 7 sessions/week of 4 min - Type: resistance training - Supervision: total (parents)	- Body weight - Calf skinfold (metal caliper) - Habitual PA (pedometer) - Physical function (maximal number of repetitions)
Grolla 2011 [23] (Italy)	Single-group intervention Study quality: poor	Adolescents/adults with PWS (N = 49, 21 F + 28 M) Data are mean (SEM) (min–max) Age, year: 23.7 (1.0) (13–42) BMI, kg/m^2^: 38.7 (1.4) (21.7–58.7) Deletion, n (%): 33 (67%)/ Uniparental disomy, n (%): 6 (12%)/ Unknown subtype, n (%): 10 (20%) GHRT: not reported	- PA program - Concurrent dietary intervention: hypocaloric diet (1500 kcal/d) - Setting: institution - Program duration: 4 weeks, repeated 4 times/y - Frequency: 6 d/week with >6 h of exercise/d - Type: aerobic training - Supervision: not reported	- Body weight and composition (DXA)
Hsu 2018 [24] (Taiwan)	Single-group intervention Study quality: fair	Adults with PWS (N = 6, 2 F + 4 M) Data are mean (SD) (min–max) Age, year: 26.1 (5.0) (20–32) BMI, kg/m^2^: mean not reported (20.7–38.4) Previously been on GHRT (N = 6)	- PA program with no concurrent dietary intervention - Setting: not reported - Program duration: 12 weeks - Frequency: 1 session/week of 120 min - Type: hand muscle strength and dexterity - Supervision: total (occupational therapists)	- Physical function (hand dynamometer, pinch gauge)
Rubin, 2019 [21] (USA)	NRCT Study quality: good	Children with PWS (N = 34, 12 F + 22 M) Data are mean (SD) Age, year: 10.8 (2.5) Body fat, %: 45.9 (10.1) GHRT: not reported	- PA program with no concurrent dietary intervention - Setting: home-based program - Program duration: 6 months - Frequency: 4 sessions/week of 25 to 45 min - Type: aerobic (playground, active video games) and resistance training - Supervision: total (parents)	- Body weight and composition (DXA) - Habitual PA (accelerometer) - Physical function (motor proficiency)
Rubin 2019 [25] (USA)	Same design as [21]	Same participants as [21] Currently on GHRT (N = 33), had previously been on GHRT (N = 9), had never been on GHRT (N = 2)	- Same intervention as the work in [21]	- Body weight and composition (DXA) - Habitual PA (accelerometer) - Blood pressure - Nutritional intake (3-day food log) - Health-related quality of life of children and parents (PedsQL 4.0)
Rubin 2018 [26] (USA)		Subsample of participants included in [21] Children with PWS (N = 18, 8 F + 10 M) Data are mean (SE) (min–max) Age, year: 10.5 (0.7) (8–16) Body fat, %: 44.6 (2.0) (26.2–55.2) Deletion, n (%): 7 (39%) Uniparental disomy, n (%): 5 (28%) Unknown subtype, n (%): 6 (33%) Currently on GHRT (N = 15), had previously been on GHRT (N = 3)	- Same intervention as the work in [21]	- Body weight and composition (DXA) - Habitual PA (accelerometer) - Nutritional intake (3-day food log) - Glucose metabolism (fasting glucose, insulin, HOMA-IR) - Lipids (TC, TG, HDL, LDL) - Inflammatory status (CRP)
Rubin 2020 [15] (USA)	Same design as [21]	Same participants as [21] Currently on GHRT (N = 33), had previously been on GHRT (N = 9), had never been on GHRT (N = 2)	- Same intervention as the work in [21]	- Body weight, body composition, bone mineral density (DXA) - Blood bone markers
Rubin 2019 [16] (USA)	Same design as [21]	Same participants as [21] GHRT: not reported	- Same intervention as the work in [21]	- Program implementation - Attrition rates, compliance, fidelity - Acceptability - Perceived difficulty, enjoyment - Barriers and facilitators
Schlumpf 2006 [22] (Switzerland)	NRCT Study quality: poor	Children with PWS (N = 7, 2 F + 5 M) Data are mean (SD) (min–max) Age, year: 8.9 (2.1) Currently or previously on GHRT (N = 7)	- PA program with no concurrent dietary intervention - Setting: home-based program - Program duration: 10 weeks - Frequency: 1 session/day of 4–10 min - Type: resistance training - Supervision: total (parents)	- Body weight and body composition (DXA) - Habitual PA (pedometer)
Shields 2020 [14] (Australia)	RCT Study quality: good	Children and adults with PWS (N = 16, 8 F + 8 M) Data are mean (SD) (min–max) Age, year: 25.0 (10.0) (13–39) BMI, kg/m^2^: 35.4 (9.4) (20.6–48.7) Deletion, n (%): 11 (69%) Uniparental disomy, n (%): 4 (25%) Unknown subtype, n (%): 1 (6%) Type 2 diabetes: 5 (31%) Currently on GHRT (N = 2)	- PA program with no concurrent dietary intervention - Setting: Community gymnasium - Program duration: 10 weeks - Frequency: 2 sessions/week of 60 min - Type: resistance training - Supervision: total (physiotherapist)	- Body weight and composition (DXA) - Muscle thickness (ultrasound images) - Muscle strength (1-RM) - Physical function (Time stairs test, Weighted box stacking test)
Shields 2020 [13] (Australia)	Same design as [14]	Same participants as [14] Currently on GHRT (N = 2)	- Same intervention as the work in [14]	- Perception of physiotherapists in-volved in the intervention (semi-structured interviews)
Vismara 2010 [19] (Italy)	NRCT (control group of healthy subjects for baseline measures) Study quality: fair	Adults with PWS (N = 11, 6 F + 5 M) Data are mean (SD) Age, year: 33.8 (4.3) BMI, kg/m^2^: 43.3 (5.9) Deletion: 10 (91%) Uniparental disomy: 1 (9%) GHRT: not reported	- PA program - Concurrent dietary intervention: hypocaloric diet (1200 kcal/d) Group 1 (N = 5) - Setting: during hospital stay - Program duration: 2 weeks - Frequency: 4 sessions/week of 60 min - Type: aerobic + resistance training - Supervision: total Group 2 (N = 6): same program followed by a home-based program - Setting: home-based program - Program duration: 6 months - Frequency: 2 sessions/week of 60 min - Type: resistance training - Supervision: none	- Physical function (isokinetic muscle strength) - Gait patterns (optoelectronic system)
Capodaglio 2011 [27] (Italy)	Same design as [19]	Same participants as [19] GHRT: not reported	- Same intervention as the work in [19]	- Postural parameters (force plate)

Articles are presented in alphabetical order and articles reporting results from the same trial are presented together.Abbreviations: 1-RM, 1-repetition maximum, an indicator of muscle strength; 6MWT, 6-min walk test; DXA, dual-energy X-ray absorptiometry; EE, energy expenditure; GHRT, growth hormone replacement therapy; HDL, high-density lipoproteins; HOMA-IR, Homeostatic Model Assessment for Insulin Resistance; NRCT, non-randomized controlled trial; NSO, non-syndromic obesity; PA, physical activity; PAEE, physical activity energy expenditure; RCT, randomized controlled trial; re; TC, total cholesterol; TG, triglycerides.

**Table 2:** Habitual PA and sedentary behavior assessed with pedometers or accelerometers in patients with PWS compared to controls.

Reference—Population	PA Volume	LPA	MVPA	Sedentary Time	Meet PA Guidelines
Compared to patients with normal weight					
Eiholzer 2003 [20]—Children	(−)				
van den Berg-Emons 2008 [31]—Children	(−)				
Compared to patients with non-syndromic obesity					
Bellicha 2020 [8]—Adults	(−)	(−)	ns*	(+)	ns
Butler 2007 [29]—Children/adults	(−)				
Castner 2014 [6]—Children		(−)	ns*	ns	
McAlister 2018 [7]—Children			(−)		ns
Rubin, 2019 [26]—Children	(−)		ns*		
Van Mil 2000 [32]—Children	(−)				
Compared to patients with another neurodevelopmental disorder (i.e., Down Syndrome, Williams Syndrome)					
Nordstrom 2013 [9]—Adults	ns*	(−)	ns	(+)	

Abbreviations: LPA, light-intensity PA; MVPA, moderate-to-vigorous PA. PA volume was assessed by daily steps, total PA whatever intensity (min/d), accelerometry counts/min, daily walking distance or total mechanical workload assessed in a calorimetric chamber. (+), higher value in the PWS group vs. the control group; (−), lower value in the PWS group vs. the control group; ns, no significant difference between group (*p* ≥ 0.15); ns*, *p*-value between 0.05 and 0.15.

**Table 3:** Effects of PA interventions on different parameters of health status.

Reference—Population	Body Weight Fat Mass	LBM	Bone Parameters	Habitual PA	Habitual Sedentary Time	Physical Function	Cardio-Metabolic Markers	QOL
PA programs								
Bellicha 2020 [8]—Adults	ns	ns		(+)	ns	(+)		ns
Eiholzer 2003 [20]—Children		(+)		(+)		(+)		
Hsu 2018 [24]—Adults						(+)		
Rubin, 2019 [21]—Children				ns	ns	(+)		
Rubin 2018 [26]—Children	ns	(+)		ns			ns	
Rubin 2019 [25]—Children	ns				ns		ns	ns
Rubin 2020 [15]—Children			(+)					
Schlumpf 2006 [22]—Children	ns	(+)		(+)				
Shields 2020 [14]—Children/adults	ns	ns				ns		
Combined PA program and dietary intervention								
Grolla 2011 [23]—Children/adults	(−)	(−)						
Vismara 2010 [19]—Adults						(+)		
Capodaglio 2011 [27]—Adults	ns					ns		

Habitual PA: MVPA [8,21], 3-d walking distance [20,22]. Physical function: walking distance [8], muscle strength [20,21] and coordination [21], handgrip strength and dexterity [24], gait parameters [19], balance capacity [27]. LBM: calf circumference [20], DXA-assessed lean body mass [14,22,23,26]. Cardiometabolic markers: decrease in IL-6 but no change in other parameters (glucose metabolism, lipids, CRP, etc.) [26]; blood pressure [25]. (+), higher value in the PWS group after vs. before PA intervention; (−), lower value in the PWS group after vs. before PA intervention; ns, no significant difference between before and after PA intervention (*p* ≥ 0.15).

## Supplementary Materials

The following are available online at https://www.mdpi.com/article/10.3390/jcm10112528/s1, Table S1: Study quality of intervention studies, Table S2: Description of habitual physical activity and sedentary behavior.
