# Supplementary material for: Physical Activity in Patients with Prader-Willi Syndrome—A Systematic Review of Observational and Interventional Studies
Source: J Clin Med. 2021 Jun 7;10(11):2528. doi: 10.3390/jcm10112528 (PMC8201387; doi:10.3390/jcm10112528)
Supplement: Supplementary file 1 [file jcm-10-02528-s001.zip › jcm-1231700-supplementary.pdf]

Systematic review

Physical activity in patients with Prader-Willi syndrome - A systematic review of observational and interventional studies

Alice Bellicha[1], Muriel Coupaye[2], Héléna Mosbah[2], Maithé Tauber[3], Jean-Michel Oppert[2], Christine Poitou[1,2*]

[1] Sorbonne University, INSERM, Nutrition and obesities: systemic approaches (NutriOmics), F-75013, Paris, France; alice.bellicha@u-pec.fr

[2] Assistance Publique-Hôpitaux de Paris (AP-HP), Pitié-Salpêrière hospital, Department of Nutrition, Rare Diseases Center of reference 'Prader-Willi Syndrome and obesity with eating disorders' (PRADORT), Sorbonne University, F-75013, Paris, France; muriel.coupaye@aphp.fr; helena.mosbah@aphp.fr; jean-michel.oppert@aphp.fr; christine.poitou-bernert@aphp.fr

[3] Centre de Référence du Syndrome de Prader-Willi, Hôpital des Enfants, Toulouse, France; Axe Pédiatrique du CIC 9302/INSERM, Hôpital des Enfants, Toulouse, France; Institut Toulousain des Maladies Infectieuses et Inflammatoires, INSERM UMR1291, CNRS UMR5051, Université Toulouse III, Toulouse, France; tauber.mt@chu-toulouse.fr

* Correspondence: christine.poitou-bernert@aphp.fr; Tel.: +33(0)142175771

SUPPLEMENTARY MATERIAL

Table S1. Study quality of intervention studies

References	Criteria														Number of "fatal flaws"	Quality rating
	1	2	3	4	5	6	7	8	9	10	11	12	13	14		
Randomized or non-randomized controlled trials																
Vismara 2010 [1]	No	NA	NR	No	No	NR	Yes	Yes	Yes	Yes	Yes	No	Yes	Yes	1	Fair
Eiholzer 2003 [2]	No	NA	NR	No	No	Yes	Yes	NA	NR	Yes	Yes	No	Yes	NR	2	Fair
Rubin, 2019 [3]	No	NA	NR	No	No	Yes	No	Yes	Yes	Yes	Yes	Yes	Yes	Yes	0	Good
Schlumpf 2006 [4]	No	NA	NR	No	No	Yes	NR	NR	NR	No	Yes	No	Yes	NR	2	Poor
Shields 2020 [5]	Yes	Yes	Yes	No	Yes	Yes	NR	NR	Yes	Yes	Yes	No	Yes	NR	0	Good
Single-group intervention studies																
Bellicha 2020 [6]	Yes	Yes	No	NR	No	Yes	Yes	No	Yes	Yes	No	NA	-	-	1	Fair
Grolla 2011 [7]	Yes	No	No	NR	Yes	No	Yes	NR	NR	No	No	NA	-	-	4	Poor
Hsu 2018 [8]	Yes	Yes	No	NR	No	Yes	Yes	NR	Yes	Yes	No	NA	-	-	1	Fair

Criteria for controlled trials: #1- Randomized study, #2- Adequate randomization method, #3- Treatment allocation concealment, #4- Blinding treatment assignment, #5- Blinding outcome assessors, #**6- Similar baseline characteristics**, #7- Drop-out rate <20%, #8- Differential drop-out rate between groups <15%, #**9- High adherence**, #**10- Similar background treatments**, #11- Valid and reliable outcome measures, #12- Sample size justification, #13- Pre-specified outcomes/subgroups, #14- All randomized participants analyzed (ITT analysis).

Criteria for single-group interventions: #1- Question/objective clearly stated, #**2- Eligibility criteria pre-defined**, #**3- Representativeness**, #4- Enrolment rates, #5- Sufficient sample size, #6- Intervention clearly described, #7- Valid and reliable outcome measures, #8- Outcome assessors blinded, #**9- Drop-out rate <20% or intent to treat analysis**, #**10- Statistical analyses examined changes in outcomes**, #11- Multiple time points for outcome measurement, #12- Account for individual changes.

Criteria defined as fatal flaws are grey shaded.

Table S2. Description of habitual physical activity and sedentary behavior

References<br>Outcomes	Patients with PWS	Control group	P-value
Bellicha 2020 [6]	**Adults with PWS**	**Adults with NSO**	
Activity counts, counts/min	Median (P25; P75): 211 (141; 333)	334 (292; 384)	**< 0.05**
Sedentary time, % wear time	72 (69; 75)	58 (53; 64)	**< 0.001**
Prolonged sedentary bouts (⩾ 30 min), % total sedentary time	41%	18%	**< 0.001**
Light - intensity PA, % wear time	24 (23; 28)	38 (32; 45)	**< 0.001**
MVPA, % wear time	1.7 (0.8; 5.0)	3.2 (2.6; 4.5)	0.11
MVPA in ⩾ 10 - min bouts, min/week	5 (0; 101)	20 (0; 68)	0.38
Meet MVPA guidelines, n (%)[1]	2 (20%)	1 (5%)	0.52
Borland 2020 [9]	**Adults with PWS**	Adults with ID: 132 (42%)	**< 0.05**
Reported participation in sports/PA over the past 3 months, %	19 (63%)	Down Syndrome: 39 (61%)	NR
		Fragile X Syndrome: 29 (56%)	NR
		Williams Syndrome: 24 (53%)	NR
		Autism: 51 (54%)	NR
Butler 2007 [10]	**Children/adults with PWS**	**Children/adults with NSO**	
PA-induced EE, kcal/min	Mean (SD): 0.86 (0.37)	1.27 (0.64)	**< 0.001**
Exercise-induced EE, kcal/min	1.92 (0.61)	3.08 (0.99)	**< 0.001**
Total mechanical work[2], W/8h	30 (12)	46 (34)	**< 0.001**
Castner 2014 [11]	**Children with PWS**	**Children with NSO**	
Sedentary time, min/d	Mean (SD): 660.5 (15.1)	638.7 (11.7)	0.26
Light-intensity PA, min/d	130.3 (9.4)	161.7 (7.0)	**< 0.01**
Moderate-intensity PA, min/d	26.2 (2.4)	28.9 (1.9)	0.36
Vigorous-intensity PA, min/d	9.9 (1.2)	14.1 (1.3)	**< 0.05**
MVPA, min/d	36.1 (3.2)	43.0 (2.9)	0.14
Meet MVPA guidelines[3], n (%)	2 (8%)	7 (18%)	NR
Duran 2016 [12]	**Children with PWS**	--	--
Light-intensity PA, min/d	Mean (SD): 129.0 (46.8)	--	--
Moderate-intensity PA, min/d	25.3 (11.2)	--	--
Vigorous-intensity PA, min/d	9.3 (5.0)	--	--
MVPA, min/d	34.6 (14.1)	--	--
Eiholzer 2003 [2]	**Children with PWS**	**Children with normal weight**	
3-days walking distance, km	Mean: 11.1	24.6	**< 0.05**
McAlister 2018 [13]	**Children with PWS**	**Children with NSO**	
MVPA, min/d	Mean (SD): 33.9 (14.7)	44.0 (17.7)	**< 0.05**
Meet MVPA guidelines[3], n (%)	1 (5%)	3 (8.8%)	ns
Nordstrom 2013 [14]	**Adults with PWS**	**Adults with Down-syndrome**	
Total PA, counts/min	Mean (SD): 249 (114.1)	306 (91.4)	0.06
Steps/d	5781 (3053)	6949 (2673)	ns
Sedentary time, min/d	564 (66;9)	491 (74.4)	**0.01**
Light-intensity PA, min/d	174 (47.5)	240 (60.3)	**< 0.001**
MVPA, min/d	26.2 (20.8)	25.5 (17.7)	ns
MVPA in ≥ 10-min bouts, min/d	14.2 (15.1)	9.4 (11.6)	ns
Meet MVPA guidelines[4], %	Females: 15%, males: 25%	Females: 8%, males: 7%	NR
Rubin, 2019 [15]	**Children with PWS**	**Children with NSO**	
Total PA, min/d	Mean (SE): 165 (11)	225 (10)	**< 0.01**
MVPA, min/d	34 (4)	45 (3)	0.07
Vigorous-intensity PA, min/d	11 (2)	14 (1)	0.22
Schlumpf 2006 [4]	**Children with PWS**	--	--
3-days walking distance, km	Mean (SD): 12.5 (6.2)	--	--
Sellinger 2006 [16]	**Children/adults with PWS**	**Down syndrome**: 1.65 (0.08)	ns
Reported frequency of PA participation[5]	Mean (SD): 1.41 (0.15)	**Williams syndrome**: 1.86 (0.09)	**< 0.05**
van den Berg-Emons 2008 [17]	**Children with PWS**	**Children without obesity**	
Duration of dynamic activities[6], % of a 24-hour period	Mean (SD): 8.7 (2.5)	12.0 (3.1)	**0.01**

Mean mobility, g	0.023 (0.004)	0.028 (0.008)	0.09
Walking speed, g	0.178 (0.018)	0.170 (0.025)	0.32
Walking periods > 10 s, n/d	137 (42)	171 (45)	0.09
Breaks in sedentary time, n/d	116 (31)	143 (42)	0.13
Van Mil 2000 [18]	**Children with PWS**	**Children with NSO**	
PA-induced EE, MJ/d	Mean (SD): 1.07 (0.7)	2.56 (1.03)	**< 0.001**
PA-induced EE relative to body weight, kJ/kg/d	23.1 (17.1)	46.09 (17.8)	**< 0.001**
PA level[7]	1.33 (0.15)	1.55 (0.12)	**< 0.001**
Woods 2018 [19]	**Adults with PWS**	--	--
Steps/d	Mean (SEM): 7632 (1171)	--	--

[1] MVPA guidelines in adults at the time of the study: 150 min/week
[2] Total mechanical work = assessed by force platforms during 8h on a calorimetric chamber
[3] MVPA guidelines in children at the time of the study: 60 min/d
[4] MVPA guidelines in adults at the time of the study: 30 min/d of MVPA in 8- to 10-min bouts.
[5] Frequency assessed with a 5-point scale: 1= rarely, never to 5= daily
[6] Walking, cycling, general movement
[7] PA level = average daily metabolic rate divided by basal metabolic rate

Abbreviations: EE, energy expenditure; ID, intellectual disabilities; MVPA, moderate-to-vigorous PA; NSO, non-syndromic obesity

References

1. Vismara, L.; Cimolin, V.; Grugni, G.; Galli, M.; Parisio, C.; Sibilia, O.; Capodaglio, P. Effectiveness of a 6-Month Home-Based Training Program in Prader-Willi Patients. *Res. Dev. Disabil.* **2010**, *31*, 1373–1379, doi:10.1016/j.ridd.2010.07.001.
2. Eiholzer, U.; Nordmann, Y.; l'Allemand, D.; Schlumpf, M.; Schmid, S.; Kromeyer-Hauschild, K. Improving Body Composition and Physical Activity in Prader-Willi Syndrome. *J Pediatr* **2003**, *142*, 73–8, doi:10.1067/mpd.2003.mpd0334.
3. Rubin, D.A.; Wilson, K.S.; Dumont-Driscoll, M.; Rose, D.J. Effectiveness of a Parent-Led Physical Activity Intervention in Youth with Obesity. *Med Sci Sports Exerc* **2019**, *51*, 805–813, doi:10.1249/MSS.0000000000001835.
4. Schlumpf, M.; Eiholzer, U.; Gygax, M.; Schmid, S.; van der Sluis, I.; l'Allemand, D. A Daily Comprehensive Muscle Training Programme Increases Lean Mass and Spontaneous Activity in Children with Prader-Willi Syndrome after 6 Months. *J Pediatr Endocrinol Metab* **2006**, *19*, 65–74.
5. Shields, N.; Bennell, K.L.; Radcliffe, J.; Taylor, N.F. Is Strength Training Feasible for Young People with Prader-Willi Syndrome? A Phase I Randomised Controlled Trial. *Physiotherapy* **2019**, doi:10.1016/j.physio.2019.01.016.
6. Bellicha, A.; Coupaye, M.; Hocquaux, L.; Speter, F.; Oppert, J.-M.; Poitou, C. Increasing Physical Activity in Adult Women with Prader-Willi Syndrome: A Transferability Study. *J Appl Res Intellect Disabil* **2020**, *33*, 258–267, doi:10.1111/jar.12669.
7. Grolla, E.; Andrighetto, G.; Parmigiani, P.; Hladnik, U.; Ferrari, G.; Bernardelle, R.; Lago, M.D.; Albarello, A.; Baschirotto, G.; Filippi, G.; et al. Specific Treatment of Prader-Willi Syndrome through Cyclical Rehabilitation Programmes. *Disabil Rehabil* **2011**, *33*, 1837–47, doi:10.3109/09638288.2010.549288.
8. Hsu, W.-L.; Chiu, V.J.-Y.; Chang, W.-H.; Lin, M.-C.; Wei, J.-T.; Tzeng, I.-S. Hand Strength and Dexterity in Patients with Prader-Willi Syndrome: A Pilot Intervention Study. *J. Int. Med. Res.* **2018**, *46*, 4669–4677, doi:10.1177/0300060518788243.
9. Borland, R.L.; Hu, N.; Tonge, B.; Einfeld, S.; Gray, K.M. Participation in Sport and Physical Activity in Adults with Intellectual Disabilities. *J. Intellect. Disabil. Res. JIDR* **2020**, *64*, 908–922, doi:10.1111/jir.12782.
10. Butler, M.G.; Theodoro, M.F.; Bittel, D.C.; Donnelly, J.E. Energy Expenditure and Physical Activity in Prader-Willi Syndrome: Comparison with Obese Subjects. *Am J Med Genet A* **2007**, *143A*, 449–59, doi:10.1002/ajmg.a.31507.
11. Castner, D.M.; Tucker, J.M.; Wilson, K.S.; Rubin, D.A. Patterns of Habitual Physical Activity in Youth with and without Prader-Willi Syndrome. *Res Dev Disabil* **2014**, *35*, 3081–8, doi:10.1016/j.ridd.2014.07.035.
12. Duran, A.T.; Wilson, K.S.; Castner, D.M.; Tucker, J.M.; Rubin, D.A. Association between Physical Activity and Bone in Children with Prader-Willi Syndrome. *J. Pediatr. Endocrinol. Metab. JPEM* **2016**, *29*, 819–826, doi:10.1515/jpem-2015-0233.
13. McAlister, K.L.; Fisher, K.L.; Dumont-Driscoll, M.C.; Rubin, D.A. The Relationship between Metabolic Syndrome, Cytokines and Physical Activity in Obese Youth with and without Prader-Willi Syndrome. *J Pediatr Endocrinol Metab* **2018**, *31*, 837–845, doi:10.1515/jpem-2017-0539.
14. Nordstrom, M.; Hansen, B.H.; Paus, B.; Kolset, S.O. Accelerometer-Determined Physical Activity and Walking Capacity in Persons with Down Syndrome, Williams Syndrome and Prader-Willi Syndrome. *Res Dev Disabil* **2013**, *34*, 4395–403, doi:10.1016/j.ridd.2013.09.021.
15. Rubin, D.A.; Duran, A.T.; Haqq, A.M.; Gertz, E.R.; Dumont-Driscoll, M. Changes in Cardiometabolic Markers in Children with Prader-Willi Syndrome and Nonsyndromic Obesity Following Participation in a Home-Based Physical Activity Intervention. *Pediatr Obes* **2018**, *13*, 734–743, doi:10.1111/ijpo.12462.
16. Sellinger, M.H.; Hodapp, R.M.; Dykens, E.M. Leisure Activities of Individuals with Prader-Willi, Williams, and Down Syndromes. *J. Dev. Phys. Disabil.* **2006**, *18*, 59–71, doi:10.1007/s10882-006-9006-8.
17. van den Berg-Emons, R.; Festen, D.; Hokken-Koelega, A.; Bussmann, J.; Stam, H. Everyday Physical Activity and Adiposity in Prader-Willi Syndrome. *J Pediatr Endocrinol Metab* **2008**, *21*, 1041–8.
18. van Mil, E.; Westerterp, K.R.; Kester, A.D.M.; Curfs, L.M.G.; Gerver, W.J.M.; Schrander-Stumpel, C.; Saris, W.H.M. Activity Related Energy Expenditure in Children and Adolescents with Prader-Willi Syndrome. *Int. J. Obes.* **2000**, *24*, 429–434, doi:10.1038/sj.ijo.0801175.

19. Woods, S.G.; Knehans, A.; Arnold, S.; Dionne, C.; Hoffman, L.; Turner, P.; Baldwin, J. The Associations between Diet and Physical Activity with Body Composition and Walking a Timed Distance in Adults with Prader-Willi Syndrome. *Food Nutr Res* **2018**, *62*, 1343–1354, doi:10.29219/fnr.v62.1343.
